# 1919. Implementation of Hydrogen Peroxide Vapor (HPV) Decontamination of N95 Respirators for Reuse During COVID-19 Pandemic

**DOI:** 10.1093/ofid/ofac492.1546

**Published:** 2022-12-15

**Authors:** Nancy L Havill, Richard A Martinello, Patrick Kenney, Cherie Bragg, Jacqueline Epright, Margaret Cintron, Italo Solla, Scott Israel

**Affiliations:** Yale New Haven Health System, New Haven, Connecticut; Yale School of Medicine, New Haven, Connecticut; Yale University, New Haven, Connecticut; Yale New Haven Health, New Haven, Connecticut; Yale New Haven Health, New Haven, Connecticut; Yale New Haven Hospital, New Haven, Connecticut; Yale New Haven Hospital, New Haven, Connecticut; YNHH, Orange, Connecticut

## Abstract

**Background:**

To ensure an adequate supply of N95 respirators in response to the global shortages caused by the COVID-19 pandemic, we evaluated and implemented hydrogen peroxide vapor (HPV) to reprocess disposable N95 respirators. Previous work performed by our team showed that HPV was effective in eradicating viable viruses from experimentally contaminated N95 respirators and that they retained their breathability and filtering efficiency for 3 cycles of HPV disinfection.

**Methods:**

A multidisciplinary team worked by performing experiments and PDSA cycles to develop the ultimate process. Key processes and stakeholders were identified and engaged in operations decisions.

**Results:**

The respirator reprocessing program was successfully implemented. One of the critical components for its success was the implementation of a Personal Protective Equipment (PPE) liaison program which was developed to create a process and local, unit-level champion for the collection of used N95s and to educate the staff on the program and provide guidance per the hospitals’ PPE policy. A courier system was implemented for the collection, transport and delivery of bulk containers of respirators between facilities. Facility Services designed and constructed a centralized respirator reprocessing center to include a receiving location, a negative pressure decontamination area to sort and stage the respirators on racks, two HPV reprocessing rooms and a clean room to receive the reprocessed N95s and to repackage and label for distribution. Standard operating procedures, staff training and competencies, and logs for documentation were created.

Within 18 weeks (March 13, 2020 through July 2020), nearly 32,000 N95 respirators were reprocessed and packaged for redistribution utilizing the 2 HPV disinfection rooms and 5 full time employees. As built, there was capacity to reprocess 5,000 respirators per day and evaluated by the U. S. Food and Drug Administration (FDA) led to the issue of an Emergency Use Authorization.

**Conclusion:**

This scalable program enabled YNHHS to ensure an adequate supply of respirators for the safety of staff during the COVID-19 pandemic and global shortages of PPE. A multidisciplinary team and leadership commitment to provide resources for space and personnel were critical for program success.

**Disclosures:**

**All Authors**: No reported disclosures.

